# The Burden of Undernutrition and Its Associated Factors Among Children Below 5 Years of Age in Bambao Region, Comoros

**DOI:** 10.3389/fnut.2022.885002

**Published:** 2022-04-26

**Authors:** Hadji Ahamada, Bruno F. Sunguya

**Affiliations:** School of Public Health and Social Sciences, Muhimbili University of Health and Allied Sciences, Dar es Salaam, Tanzania

**Keywords:** undernutrition, stunting, wasting, underweight, under-five, Comoros

## Abstract

**Background:**

Undernutrition remains a major public health problem in low- and middle-income countries and Comoros is no exception. This study aimed to examine the prevalence and identify the risk factors of undernutrition among children under-five years in Bambao region, Comoros.

**Methods:**

This cross-sectional study was conducted in Bambao region among 837 under-five years and their caregivers. Analyses were conducted using both descriptive and logistic regression to examine the magnitude and factors associated with stunting, wasting and underweight.

**Results:**

Prevalence of stunting, wasting and underweight were 21.6, 13.7, and 13.6% respectively. Factors associated with stunting were caregiver's secondary education level compared to no education (AOR = 1.89, 95% CI: 1.04–3.43, *P* < 0.04), age of child between 13–24 months compared to 0–12 months (AOR = 2.69, 95% CI: 1.44–5.01, *P* < 0.001), and food insecurity (AOR = 2.55, 95% CI: 1.20–5.41, *P* < 0.02). Children aged 25–59 months were 78% less likely to have wasting compared to those with 0–12 months (AOR = 0.22, 95% CI: 0.10–0.51, *P* < 0.001). Wasting was also associated with food insecurity (AOR = 2.70, 95% CI: 1.12–6.49, *P* < 0.03), and low birthweight (AOR = 3.21, 95% CI: 1.73–5.94, *P* < 0.001). Children aged between 25–59 months were 86% less likely to have underweight compared to those aged 0–12 months (AOR = 0.14, 95% CI: 0.06–0.36, *P* < 0.001). Food insecurity (AOR = 2.65, 95% CI: 1.08–6.54, *P* < 0.03), low birthweight (AOR = 3.15, 95% CI: 1.67–5.93, *P* < 0.001), and non-exclusively breastfeeding (AOR = 2.37, 95% CI: 1.15–4.90, *P* < 0.02) were also associated with underweight.

**Conclusion:**

More than one in five children under-five is stunted in Bambao region, Comoros. Moreover, more than 13% are underweight or wasted calling for streamlined efforts to address poor feeding practices, food insecurity, low birthweight, and socio-demographic disadvantages in this and other areas with similar context.

## Introduction

Childhood undernutrition is one of the global leading cause of under-five mortality and morbidity (1, 2), contributing to more than a third of 5.4 million preventable children deaths annually (3). Stunting, underweight, and wasting have significant short and long-term consequences on child growth, health, and development (4, 5). They include increased diseases severity (6), delayed physical and mental development (7), poor academic performance (8) and premature death (9). A child is considered to be stunted, wasted, or underweight when their height-for-age, weight-for-height, and weight-for-age, respectively, is minus two standard deviations below the median population (10, 11).

About 144 million children under-five years of age were stunted, while 47 million suffered from wasting, and 99 million were underweight in 2020 (12). Such unprecedented burden varies with regions, with African continent suffering the biggest brunt of the burden with 39.4% of stunting, 24.9% of underweight, and 10.3% of wasting among children under-five years of age (13–15). Although the prevalence of stunting among children under-five years decreased between 2000 and 2016, from 38 to 23%, the number of affected children had increased from 50.6 to 58.7 million owing to population growth (16).

Evidence suggested a multifactorial causes of undernutrition among children under-fives (1, 17). They ranged from basic causes such as poverty and other economic and demographic disadvantages (18); underlying factors such as food insecurity and poor water supply (19), access to health services (20); and immediate factors such as poor dietary patterns (2), childhood illnesses, among others. Such factors vary between and within countries and therefore call for local studies to ensure tailored interventions that are informed by evidence.

The burden of child undernutrition is not different in Comoros compared to other African countries (21, 22). Evidence from a nationally representative survey conducted a decade ago showed stunting, underweight, and wasting to be prevalent among 31.1, 16.9, and 11.3% of children under-fives. The recent national report indicated a decline of stunting to 22.6% (9, 22, 23). Evidence is scanty on the magnitude of undernutrition and its risk factors among children below 5 years in different regions (9, 24). Such evidence is important to design context-appropriate strategies to mitigate the unprecedented burden. Therefore, this study aimed to assess the prevalence of undernutrition and its associated factors among under-five children in Bambao region, Comoros.

## Materials and Methods

### Study Design and Setting

This cross-sectional study was conducted among children under-five years and their caregivers in Bambao region, Comoros in July 2021. The Union of Comoros consists of three main islands, which are Ngazidja, Anjouan and Moheli. About 75% of the population of 882,605 people lives in rural areas (25). Bambao region has a population of 73,000 people. The region is made of sixteen villages along the sea and the mountainous areas. Most of Bambao is sub-urban with farmlands and forests (26).

### Study Population

The selected participants included children aged below 5 years and their caregivers who lived in Bambao region. The study excluded children with critical illnesses or disabilities.

### Sample Size Determination

The sample size was determined using a single population proportion formula: *n* = Z^2^ P(1-P)/E^2^ (3). The national representative sample indicated the prevalence of 31.1% for stunting, wasting (11.3%) and underweight (16.9%) (9, 23). The standard normal deviate of 1.96 on using 95% confidence interval (Z) and 5% margin of error (E) were used, and gave a minimum sample size of 329. A design effect of 2, and 10% non-response (refusal or closed houses after three repeated visit) were considered (3). The minimum sample size was therefore 730.

### Sampling Procedures and Technique

**Figure 1:**
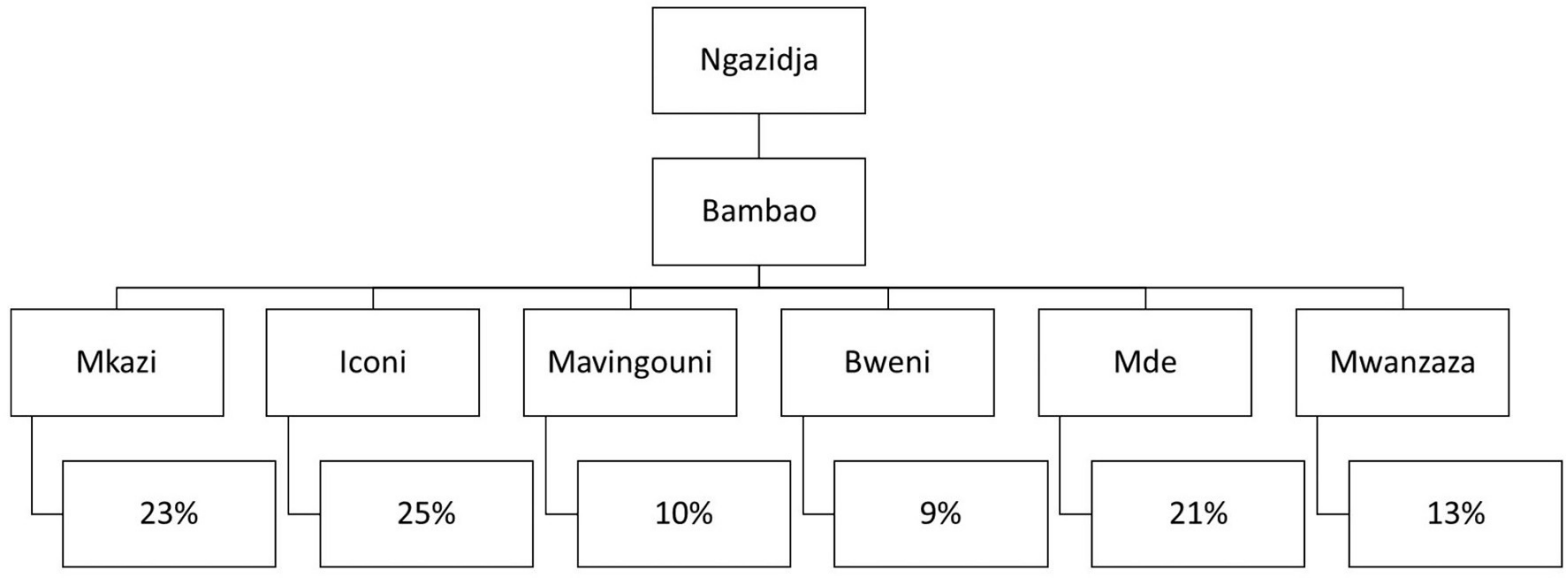
Recruitment pattern of the sample in respective households.

A multistage cluster sampling technique was used to enroll the study participants from the communities. First, the Bambao region was selected out of the seven regions in Ngazidja using simple random sampling (lottery) method. Second, six out of sixteen villages were similarly selected. Third, the total number of children under-five years in the selected villages was taken from the respective households using the registration at health posts. Then, the calculated sample size *n* = 730 was proportionally distributed to the selected villages based on the total number of households with under-five years children in each village. Finally, participants were selected using systematic random sampling technique after identifying the first household randomly in each village and proceed to the second participant based on the K^th^ interval (*K* = 3) (Figure 1). Whenever there were two or more children under-five years in a household, the youngest child was selected to avoid recall bias.

### Study Variables and Their Measurements

The outcome variables were stunting, wasting, and underweight. All children below−2SD of height-for-age Z-score (HAZ), weight-for-age Z-score (WAZ) and weight-for-height Z-score (WHZ) for the reference population were considered stunted, underweighted and wasted respectively (27).

Independent variables included socio-demographic characteristics (age of child, child sex, age of mother/caregiver, family size, maternal/caregiver education level, household occupation, marital status of the mother and household wealth index); child characteristics (weight at birth, child feeding practices, childhood diseases/history of illness, feeding frequency, dietary diversity, and child immunization); and household characteristics (household food insecurity, main source of food, main source of water and antenatal care visit).

Child and caregiver's variables had their questions extracted from the Comorian Demographic and Health Survey 2012–2013 of women and children questionnaires. Education level was categorized into no formal education, primary level education, secondary level education and above secondary level education including college and others.

For feeding practices, exclusive breastfeeding was assessed through asking mothers/caregivers to recall on the period the child was exclusively breastfed as recommended by WHO (28). Responses were categorized into exclusively breastfed, or non-exclusively breastfed. Exclusive breastfeeding is defined as the practice of giving an infant breast-milk only for the first six months of life (28, 29). Complementary feeding referred to the time when introduction of soft, semi-solid and solid food item in addition to breast milk was done (30). Feeding frequency assessment was done by asking the mothers/caregivers to recall the number of times they fed/nourished their children in the previous 24 h as applied in others studies (31). A list of common food in Comoros was used to assess consumption of each of eight food groups provided by Food and Nutrition Technical Assistance (FANTA) and used to calculate the child Dietary Diversity Score (DDS) (32). The DDS ranged between 0–8 and consumption of food from at least four food groups means that the child has a high likelihood of consuming at least one animal source of food and at least one fruit or vegetable in addition to a staple food (grains, roots or tubers).

History of illness included malaria, fever, skin disease, oral disease, acute respiratory infections, pneumonia, vomiting, or diarrhea in the past 1 month as stated in previous studies (31). The responses were categorized into Yes or No. Child immunization, was defined as full immunized or not completed vaccination based on the United Nations International Children's Emergency Fund (UNICEF) guideline (11). Information was obtained from child's clinic cards. Antenatal Care visits during pregnancy of the child were categorized into three or less visits as low visit, or four and above visits as required number of visit recommended by the World Health Organization (WHO) (29). Birthweight of the child was categorized into below 2.5kg as low birthweight, between 2.5kg to 3.5kg as normal or above 3.5kg considered big baby (29). Household food insecurity access scale (HFIAS) was assessed using nine item questionnaires provided by FANTA (33). The scores were calculated according to the HFIAS Indicator guide. Generally, the scores are grouped into four categories; food secure, mildly insecure, moderately insecure and severely food insecure (33). In this study the mild, moderate and severe were collapsed into one group which resulted in two categories of HFIAS.

Household wealth index, was derived from household's possession, consumer goods or durable assets they own, ranging from a television to a bicycle or car, housing characteristics, such as source of drinking water, toilet facilities, and flooring materials which were adapted from previous study (34). Scores were derived from principal component analysis (PCA) using SPSS. There were 34 initial variables taken into principle component analysis in the SPSS, which were then iteratively reduced into 9 components. The first output component was selected as the wealth index as it showed the highest variation of 35% with a Kaiser-Meyer Measure Sampling adequacy of 0.758 which is considered acceptable. The wealth index for each household was weighted and then ranked before creating three equal wealth terciles. These were categorized as poor, middle and rich (4). For household economic activities, mothers**/**caretakers were asked to self-report the main occupation of the head of household. Responses were based on the main economic activities common in the area. There were four categories of occupation: farming, employees, businessman/woman, unskilled manual labor and unemployed. These were then classified as employed, self-employed and unemployed.

### Data Collection Tools and Procedure

Quantitative data were collected using structured questionnaire. The tool was adopted from Comorian Demographic and Health Survey (CDHS) 2012/13 for household, caregivers and child factors (23). Mothers/caregivers were requested to provide consent to participate in the study. Interviews were conducted in privacy to enable respondents provide information. Field data collection was conducted in 4 weeks from 4th to 29th July 2021. Socio-demographic data of mothers/caregivers, child characteristics and household characteristics were collected using face-to-face interview. The questionnaire was prepared in English translated into Shi-Comore and administered by trained research assistants and principal investigator for data collection. An electronic data entry form was created using Kobo toolbox and used via smart phones for data collection. Before interview, the objective of the study was explained to study participants and it was guaranteed that the information will be kept confidential; then a written informed consent was obtained from mothers or caregivers. To ensure data quality, 2 days training were given to data collectors by principal investigator. The weight and height/length of all children were measured using the standard anthropometric measurement protocol designed by Food and Nutrition Technical Assistance project in 2007 (35). For children <2 years, weight were measured using Salter hanging weight scale and height was measured in recumbent position using standard measuring board calibrated in centimeters (36). Weight for children older than 2 years was measured using SECA digital weight scale to the nearest 0.1 kg and height was measured in standing position to the nearest 1 cm using UNICEF wooden height board (10, 37). Calibration for weight and height instruments was done upon every case examination. All measurements were taken twice and the mean values were used for data analysis. The child's age was recorded in months by observing the child's birth certificate or vaccination cards.

### Data Entry and Analysis

Analyses were conducted using both descriptive and logistic regression methods. Descriptive statistics were used to estimate burden and assess characteristics of different forms of undernutrition. Anthropometric data were computed using Emergency Nutrition Assessment (ENA) 2007 software into Height-for-Age Z-scores (HAZ), Weight-for-Height Z-scores (WHZ) and Weight-for-Age Z-scores (WAZ) taking sex into consideration. The WHO classification was used to classify the nutritional status of the children (10). Both bivariate and multiple logistic regression analysis were used to determine risk factors of undernutrition (stunting, wasting and underweight). All variables found to be associated to undernutrition in bivariate analysis using Crude Odds Ratio with 95% CI at significant level of ≤ 0.2 were taken to multiple logistic regression analysis in order to control confounders. Statistical association between dependent and independent variables were declared significant at *p-*value of ≤ 0.05.

## Results

### Participants' Characteristics

Data of 837 children-caregivers' pairs were finally analyzed. Responding caregivers had a mean age of 29.5 (SD: 6.48), and the majority were the mothers (91.9%), married (88.2%) and had at least primary level education (74.7%). Most of the households were food secure (81.6%) and the main source of food was from purchasing (97.4%).

Most households (80.3%) had a dietary diversity more than four score (≥4) (Table 1). In terms of child feeding practices, nearly all children were breastfed (98.8%) and about half of them were exclusively breastfed during the first 6 months of life (53.2%) and nearly all children stopped breastfeeding before 24 months (99.7%). For most children, feeding frequency was <4 times per day (65%) (Supplementary Table 1). In terms of healthcare characteristics, most mothers attended antenatal care visits more than four times (62.4%) and nearly all children were born in a health facility (*n* = 98%). Most children completed vaccination or were on routine vaccination (95.2%). Around 54% of children had suffered any type of illnesses, most commonly fever (20.3%), cough (16.7%), diarrhea (12.8%) and malaria (8.1%). Boys had significantly more malaria episodes than girls (Supplementary Table 2).

**Table 1:** Socio-demographic and household characteristics of children aged 0–59 months in Bambao region, July 2021 (*N* = 837).

**Variables**	**Total**		**Male**		**Female**		***p*-value**
	***N***	**%**	***N***	**%**	***N***	**%**	
**Age of child (months)**						
0–12	195	23.3	97	25.5	98	21.5	0.161
13–24	186	22.2	90	23.6	96	21.1	
25–59	456	54.5	194	51.1	262	57.33	
**Child relationship to caregiver**						
Mother	769	91.9	353	92.9	416	91.0	0.360
Father	17	2.0	7	1.8	10	2.2	
Grandmother	22	2.6	6	1.6	16	3.5	
Relative	29	3.5	14	3.7	15	3.3	
**Age of caregiver (Years)**						
0–19	40	4.8	17	4.5	23	5.0	0.741
20–29	397	47.4	186	48.8	211	46.3	
≥30	400	47.8	178	46.7	222	48.7	
**Child birthweight in Kg (*****n*** **=** **801)**						
<2.5	126	15.7	59	16.2	67	15.4	0.693
2.5–3.5	639	79.8	292	80.0	347	79.6	
>3.5	36	4.5	14	3.8	22	5.0	
**Marital status of caregiver**						
Married	738	88.2	331	86.9	407	89.3	0.289
Not married	99	11.8	50	13.1	49	10.7	
**Caregiver**'**s education level**						
No education	212	25.3	101	26.6	111	24.3	0.373
Primary education	226	27.0	108	28.4	118	25.8	
Secondary education	309	36.9	128	33.7	181	39.6	
Post-secondary education	90	10.8	43	11.3	47	10.3	
**Household occupation**						
Employed	364	43.5	158	41.5	206	45.2	0.558
Self-employed	445	53.2	210	55.1	225	51.5	
Not employed	28	3.3	13	3.4	15	3.3	
**Household food insecurity**						
Food secure	683	81.6	305	80.1	378	82.9	0.291
Food insecure	154	18.4	76	19.9	78	17.1	
**Weighted wealth index**						
Poor	265	31.7	127	33.3	138	30.3	0.278
Middle	292	34.9	122	32.0	170	37.3	
Rich	280	33.5	132	34.6	148	32.5	
**Family size**						
<5 people	609	72.8	282	74.0	327	71.7	0.456
≥5 people	228	27.2	99	26.0	129	28.3	
**Main source of food**						
Purchasing	815	97.4	370	97.1	445	97.6	0.669
Own crop farming	22	2.6	11	2.9	11	2.4	

### Burden of Undernutrition

**Figure 2:**
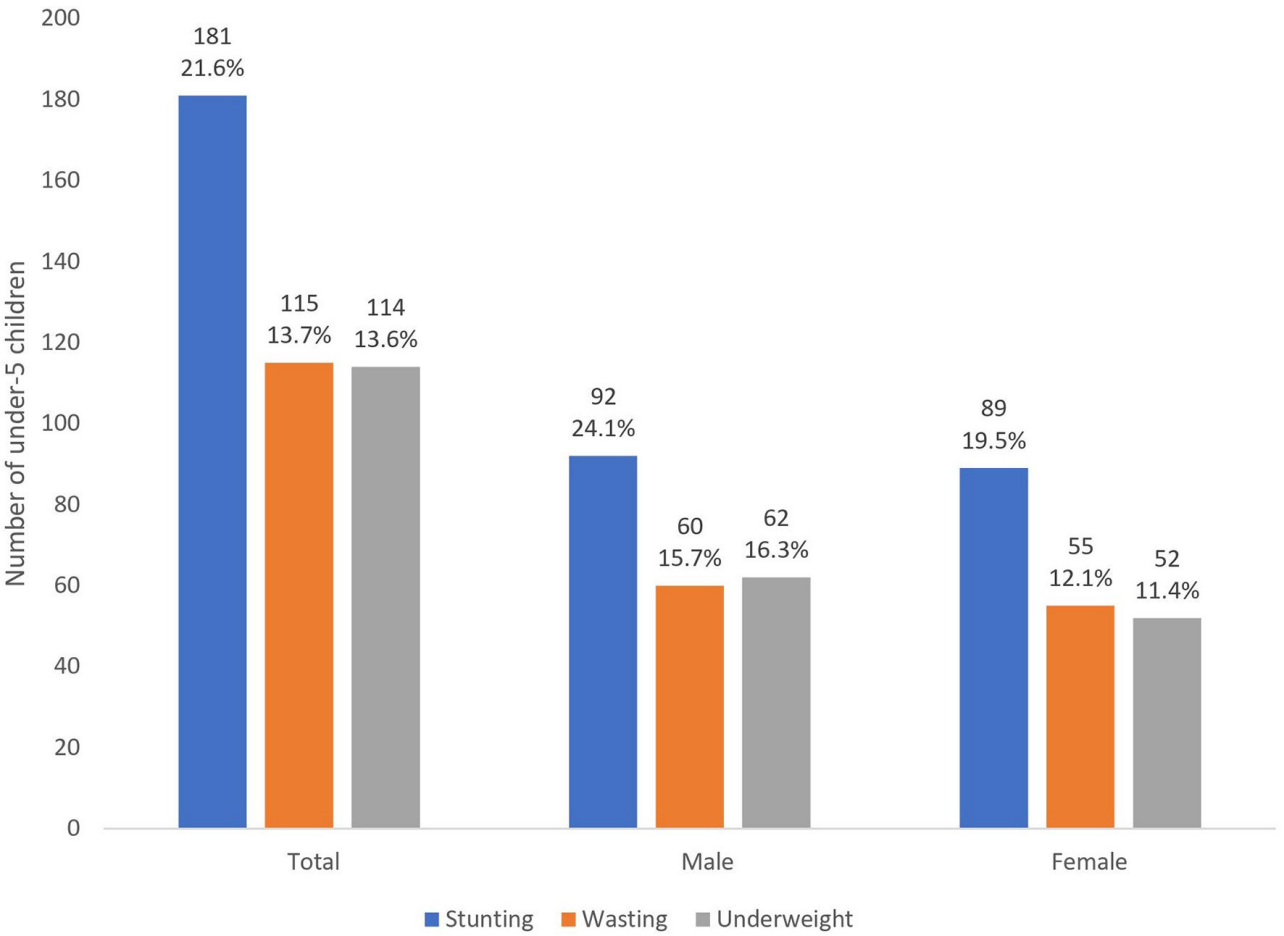
Nutrition status among children aged 0–59 months in Bambao region, July 2021 (*N* = 837).

The prevalence of stunting, wasting and underweight was 21.6, 13.7, and 13.6%, respectively. The burden varied with child's sex, with higher prevalence among boys compared to girls. All forms were higher among boys (24.1% stunting, 15.7% wasting and 16.3% underweight) compared to girls (19.5% stunting, 12.1% wasting and 11.4% underweight). However, the difference did not reach a statistical significant level except for underweight (*P* = 0.041) (Figure 2).

### Determinants of Stunting

After adjusting for covariates and confounders, children between 13–24 months were nearly three times more likely to be stunted compared to children 0–12 months, (AOR: 2.69, 95% CI 1.44–5.01). Also, food insecurity significantly increased the odds of stunting three-folds compared to being food-secure (AOR: 2.55, 95% CI 1.20–5.41). Children whose caregiver had secondary education were at increased odds of stunting in comparison to those who have no education (AOR: 1.89, 1.04–3.43) (Table 2).

**Table 2:** Bivariate and multiple logistic regression for factors associated with stunting (HAZ < −2SD) among children age 0–59 months in Bambao region, July 2021 (*N* = 837).

**Variable**	**Stunting**		**Bivariate logistic regression**			**Multiple logistic regression**		
	**No**	**Yes**	**COR**	**95% CI**	***p-*value**	**AOR**	**95% CI**	***p-*value**
**Age of child (months)**								
0–12	148 (75.9)	47 (24.1)						
13–24	123 (66.1)	63 (33.9)	1.61	1.03–2.52	0.04	2.69	1.44–5.01	<0.001^*^
25–59	385 (84.4)	71 (15.6)	0.58	0.84–0.88	0.01	1.27	0.65–2.47	0.48
**Sex of child**								
Female	367 (80.5)	89 (19.5)						
Male	289 (75.9)	92 (24.1)	1.31	0.94–1.83	0.11	1.25	0.87–1.78	0.23
**Marital status of caregiver**								
Married	587 (79.5)	151 (20.5)						
Not married	69 (69.7)	30 (30.3)	1.69	1.06–2.69	<0.001	1.20	0.69–2.09	0.52
**Caregiver**'**s education level**								
No education	173 (81.6)	39 (18.4)						
Primary education	165 (73.0)	61 (27.0)	1.64	1.04–2.59	0.03	1.75	0.98–3.13	0.06
Secondary education	247 (79.9)	62 (20.1)	1.11	0.71–1.74	0.64	1.89	1.04–3.43	0.04^*^
Post-secondary education	71 (78.9)	19 (21.1)	1.19	0.64–2.19	0.58	1.94	0.90–4.19	0.09
**Occupation of head of household**								
Employed	291 (79.9)	73 (20.1)						
Self-employed	345 (77.5)	100 (22.5)	1.16	0.82–1.62	0.40			
Unemployed	20 (71.4)	8 (28.6)	1.60	0.68–3.77	0.29			
**Weighted wealth index**								
Poor	199 (75.1)	66 (24.9)						
Middle	235 (80.5)	57 (19.5)	0.731	0.49–1.09	0.13	0.87	0.48–1.59	0.65
Rich	222 (79.3)	58 (20.7)	0.79	0.53–1.18	0.24	0.99	0.50–1.93	0.97
**Child ever breastfed**								
No	6 (60.0)	4 (40.0)						
Yes	650 (78.6)	177 (21.4)	0.41	0.11–1.46	0.53			
**Breastfeeding initiation time**								
Within an hour	395 (79.2)	104 (20.8)						
After 1 h	261 (77.2)	77 (22.8)	1.12	0.80–1.56	0.50			
**Exclusive breastfeeding**								
Exclusively breastfed	368 (84.2)	69 (15.8)						
Non-exclusively breastfed	277 (71.9)	108 (28.1)	2.08	1.48–2.92	<0.001	1.23	0.77–1.98	0.39
**Child feeding frequency (24 h)**								
<4 times	436 (80.0)	109 (20.0)						
4 times and more	220 (75.3)	72 (24.7)	1.31	0.93–1.84	0.12	1.30	0.77–2.19	0.32
**Dietary diversity score**								
<3 scores	112 (67.9)	53 (32.1)						
4 scores or more	544 (81.0)	128 (19.0)	0.5	0.34–0.73	<0.001	0.88	0.46–1.66	0.69
**Child vaccination**								
Completed vaccination	519 (80.5)	126 (19.5)						
Not completed vaccination	32 (80.0)	8 (20.0)	1.03	0.46–2.9	0.94	0.44	0.14–1.33	0.14
On vaccination routine	105 (69.1)	47 (30.9)	1.84	1.24–2.74	0.002	1.15	0.57–2.32	0.70
**Child illness**								
No	369 (81.8)	82 (18.2)						
Yes	287 (74.4)	99 (25.6)	1.55	1.12–2.16	<0.001	1.49	0.98–2.28	0.06
**Antenatal care visit**								
<3 times	13 (86.7)	2 (13.3)						
4 times and more	616 (77.9)	175 (22.1)	1.85	0.41–8.26	0.42			
**Child birthweight**								
Between 2.5–3.5kg	510 (79.8)	129 (20.2)						
Below 2.5kg	84 (66.7)	42 (33.3)	2.00	1.30–3.00	0.001	1.11	0.66–1.88	0.69
Above 3.5kg	31 (86.1)	5 (13.9)	0.64	0.24–1.67	0.36	0.54	0.19–1.49	0.23
**Food insecurity access scale**								
Food secure	560 (82.0)	123 (18.0)						
Food insecure	96 (62.3)	58 (37.7)	2.75	1.88–4.02	<0.001	2.55	1.20–5.41	0.02^*^
**Main source of food**								
Purchasing	641 (78.7)	174 (21.3)						
Own crop farming	15 (68.2)	7 (31.8)	1.72	0.69–4.28	0.25			

* *Significant at p-value < 0.05*.

*COR, Crude Odds Ratio; AOR, Adjusted Odds Ratio; CI, Confident Interval; SD, Standard Deviation; HAZ, Height-for-Age Z-score*.

### Determinants of Wasting

Low birthweight (below 2.5kg) significantly increased the risk of wasting (AOR: 3.21, 95% CI 1.73–5.94). Food insecurity was associated with a three-fold increase in the risk of wasting (AOR: 2.85, 95% CI 1.17–6.94). Children aged 25–59 months were 78% less likely to be wasted compared to children 0–12 months old (AOR: 0.22, 95% CI 0.10–0.51) (Table 3).

**Table 3:** Bivariate and multiple logistic regression for factors associated with wasting (WHZ < −2SD) among children age 0–59 months in Bambao region, July 2021 (*N* = 837).

**Variable**	**Wasting**		**Bivariate logistic regression**			**Multiple logistic regression**		
	**No**	**Yes**	**COR**	**95% CI**	***p-*value**	**AOR**	**95% CI**	***p-*value**
**Age of child (months)**							
0–12	130 (66.7)	65 (33.3)						
13–24	153 (82.3)	33 (17.7)	0.43	0.27–0.70	0.001	0.76	0.37–1.55	0.45
25–59	439 (96.3)	17 (3.7)	0.077	0.04–0.14	<0.001	0.22	0.10–0.51	<0.001^*^
**Sex of child**							
Female	401 (87.9)	55 (12.1)						0.43
Male	321 (84.3)	60 (15.7)	1.36	0.92–2.02	<0.001	1.23	0.74–2.04	
**Marital status of caregiver**							
Married	645 (87.4)	93 (12.6)						
Not married	77 (77.8)	22 (22.2)	1.98	1.18–3.34	0.01	0.66	0.30–1.48	0.31
**Caregiver**'**s education level**							
No education	182 (85.8)	30 (14.2)						
Primary education	173 (76.5)	53 (23.5)	1.86	1.13–3.05	0.01	1.71	0.85–3.45	0.13
Secondary education	286 (92.6)	23 (7.4)	0.49	0.28–0.87	0.01	1.11	0.50–2.47	0.80
Post-secondary education	81 (90.0)	9 (210.0)	0.67	0.31–1.49	0.67	1.32	0.45–3.90	0.61
**Occupation of head of household**							
Employed	331 (90.9)	33 (9.1)						
Self-employed	373 (83.9)	72 (16.2)	1.94	1.25–3.00	0.003	1.01	0.53–1.92	0.98
Unemployed	18 (64.3)	10 (35.7)	5.57	2.38–13.06	<0.001	0.82	0.22–3.10	0.77
**Weighted wealth index**							
Poor	197 (74.3)	68 (25.7)						
Middle	259 (88.7)	33 (11.3)	0.37	0.23–0.58	<0.001	0.53	0.26–1.10	0.09
Rich	266 (95.0)	14 (5.0)	0.15	0.08–0.28	<0.001	0.69	0.27–1.76	0.44
**Child ever breastfed**							
No	7 (70.0)	3 (30.0)						
Yes	715 (86.5)	112 (13.5)	0.37	0.09–1.43	0.22			
**Breastfeeding initiation time**							
Within an hour	454 (91.0)	45 (9.0)						
After 1 hr	268 (79.3)	70 (20.7)	2.64	1.76–3.95	<0.001	1.37	0.73–2.57	0.33
**Exclusive breastfeeding**							
Exclusively breastfed	412 (94.3)	25 (5.7)						
Non-exclusively breastfed	298 (77.4)	87 (22.6)	0.21	0.13–0.33	<0.001	1.11	0.57–2.15	0.76
**Child feeding frequency (24 h)**							
<4 times	491 (90.1)	54 (9.9)						
4 times and more	231 (79.1)	61 (20.9)	2.40	1.61–3.57	<0.001	0.74	0.37–1.51	0.41
**Dietary diversity score**							
<3 scores	103 (62.4)	62 (37.6)						
4 scores or more	619 (92.1)	53 (7.9)	0.14	0z.09–0.22	<0.001	0.79	0.38–1.66	0.53
**Child vaccination**							
Completed vaccination	597 (92.6)	48 (7.4)						
Not completed vaccination	29 (72.5)	11 (27.5)	4.72	2.22–10.03		1.09	0.37–3.27	0.87
On vaccination routine	96 (63.2)	56 (36.8)	7.26	4.67–11.28	<0.001	1.29	0.56–2.97	0.55
**Child illness**							
No	406 (90.0)	45 (10.0)						
Yes	316 (81.9)	70 (18.1)	2.00	1.34–3.00	<0.001	1.74	0.95–3.18	0.07
**Antenatal care visit**							
<3 times	8 (53.3)	7 (46.7)						
4 times and more	692 (87.5)	99 (12.5)	0.16	0.06–0.46	0.001	0.63	0.13–2.95	0.55
**Child birth weight**							
Between 2.5–3.5 kg	589 (92.2)	50 (7.8)						
Below 2.5 kg	78 (61.9)	48 (38.1)	7.25	4.57–11.50		3.21	1.73–5.94	<0.001^*^
Above 3.5 kg	29 (80.6)	7 (19.4)	2.84	1.19–6.82	<0.001	2.60	0.94–7.19	0.07
**Food insecurity access scale**							
Food secure	636 (93.1)	47 (6.9)						
Food insecure	86 (55.8)	68 (44.2)	10.70	6.93–16.53	<0.001	2.70	1.12–6.49	0.03^*^
**Main source of food**							
Purchasing	708 (86.9)	107 (13.1)						
Own crop farming	14 (63.6)	8 (36.4)	3.78	1.55–9.23	0.003	1,28	0.34–4.87	0.72

* *Significant at p-value < 0.05*.

*COR, Crude Odds Ratio; AOR, Adjusted Odds Ratio; CI, Confident Interval; SD, Standard Deviation; WHZ, Weight-for-Height Z-score*.

### Determinants of Underweight

In the multivariate analysis, children aged 25–59 months were 86% less likely to have wasting compared to children 0–12 months old (AOR: 0.14, 95% CI 0.06–0.36). Food insecurity was more likely to double the risk of underweight (AOR: 2.65, 95% CI 1.08–6.54), while Low birthweight significantly increased the risk of underweight (AOR: 3.15, 95% CI 1.67–5.93). In addition, non-exclusive breastfeeding in the first six months was significantly associated with underweight (AOR: 2.37, 95% CI 1.15–4.90) (Table 4).

**Table 4:** Bivariate and multiple logistic regression for factors associated with underweight (WAZ < −2SD) among children age 0–59 months in Bambao region, July 2021 (*N* = 837).

**Variable**	**Underweight**		**Bivariate logistic regression**			**Multiple logistic regression**		
	**No**	**Yes**	**COR**	**95% CI**	***p-*value**	**AOR**	**95% CI**	***p-*value**
**Age of child (months)**							
0–12	128 (65.6)	67 (34.4)						
13–24	150 (80.6)	36 (19.4)	0.46	0.29–0.73	0.001	0.79	0.38–1.64	0.53
25–59	445 (97.6)	11 (2.4)	0.05	0.02–0.09	<0.001	0.14	0.06–0.36	<0.001^*^
**Sex of child**							
Female	404 (88.6)	52 (11.4)						
Male	329 (83.7)	62 (16.3)	1.51	1.02–2.25	<0.001	1.33	0.77–2.30	0.30
**Marital status of caregiver**							
Married	644 (87.3)	94 (12.7)	1.73	10.2–2.97	0.04	0.78	0.34–1.76	0.55
Not married	79 (78.9)	20 (20.2)						
**Caregiver**'**s education level**							
No education	182 (85.8)	30 (14.2)						
Primary education	172 (76.1)	54 (23.9)	1.91	1.16–3.12	0.01	2.14	1.01–4.53	0.05
Secondary education	286 (92.6)	23 (7.4)	0.49	0.28–0.87	0.014	1.84	0.79–4.27	0.16
Post-secondary education	83 (92.2)	7 (7.8)	0.51	0.22–1.21	0.13	1.31	0.39–4.41	0.66
**Occupation of head of household**							
Employed	336 (92.3)	28 (7.7)						
Self-employed	367 (82.5)	78 (17.5)	2.55	1.62–4.03	<0.001	1.22	0.61–2.44	0.57
Unemployed	20 (71.4)	8 (28.6)	4.80	1.94–11.88	<0.001	0.48	0.11–2.06	0.32
**Weighted wealth index**							
Poor	198 (74.7)	67 (25.3)						
Middle	256 (87.7)	36 (12.3)	0.42	0.27–0.65	<0.001	0.57	0.27–1.22	0.15
Rich	269 (96.1)	11 (3.9)	0.12	0.06–0.24	<0.001	0.46	0.17–1.27	0.14
**Child ever breastfed**							
No	7 (70.0)	3 (30.0)						
Yes	716 (86.6)	111 (13.4)	0.36	0.92–1.42	0.20			
**Breastfeeding initiation time**							
Within an hour	455 (91.2)	44 (8.8)						
After 1 h	268 (79.3)	70 (20.7)	2.70	1.80–4.05	<0.001	1.64	0.84–3.22	0.15
**Exclusive breastfeeding**							
Exclusively breastfed	205 (37.3)	344 (62.7)						
Non-exclusively breastfed	232 (85.0)	41 (15.0)	8.62	4.97–14.95	<0.001	2.37	1.15–4.90	0.02^*^
**Child feeding frequency (24 h)**							
<4 times	492 (90.3)	53 (9.7)						
4 times and more	231 (79.1)	61 (20.9)	2.45	1.64–3.66	<0.001	0.70	0.33–1.50	0.36
**Dietary diversity score**							
<3 scores	98 (59.4)	67 (40.6)						
4 scores or more	625 (93.0)	47 (7.0)	0.11	0.07–0.17	<0.001	0.70	0.32–1.50	0.36
**Child vaccination**							
Completed vaccination	598 (92.7)	47 (7.3)						
Not completed vaccination	28 (70.0)	12 (30.0)	5.45	2.6–11.41	<0.001	1.16	0.35–3.83	0.80
On vaccination routine	97 (63.8)	55 (36.2)	7.21	4.63–11.25	<0.001	0.91	0.37–2.22	0.84
**Child illness**							
No	403 (89.4)	48 (10.6)						
Yes	320 (82.9)	66 (17.1)	1.73	1.16–2.58	0.007	1.44	0.75–2.75	0.27
**Antenatal care visit**							
<3 times	9 (60.0)	6 (40)						
4 times and more	692 (87.5)	99 (12.5)	0.22	0.075–0.62	0.004	1.00	0.19–5.38	1.00
**Child birthweight**							
Between 2.5–3.5kg	589 (92.2)	50 (7.8)						
Below 2.5kg	74 (58.7)	52 (41.3)	8.28	5.24–13.08	<0.001	3.15	1.67–5.93	<0.001^*^
Above 3.5kg	33 (91.7)	3 (8.3)	1.07	0.32–3.62	0.91	0.72	0.19–2.80	0.63
**Food insecurity access scale**							
Food secure	641 (93.9)	42 (6.1)						
Food insecure	82 (53.2)	72 (46.8)	13.40	8.59–20.90	<0.001	2.65	1.08–6.54	0.03^*^
**Main source of food**							
Purchasing	708 (86.9)	107 (13.1)						
Own crop farming	15 (68.2)	7 (31.8)	3.09	1.23–7.45	0.02	0.82	0.19–3.47	0.78

* *Significant at p-value < 0.05*.

*COR, Crude Odds Ratio; AOR, Adjusted Odds Ratio; CI, Confident Interval; SD, Standard Deviation; WAZ, Weight-for-Age Z-score*.

## Discussion

This study assessed the prevalence of child undernutrition and its associated factors in an urban setting in Comoros. Although the magnitudes are slightly lower than the nationally representative survey conducted a decade ago, the current magnitudes remains unprecedently high at 21.6, 13.7, and 13.6% for stunting, wasting and underweight respectively (9, 23). The progress is therefore insufficient to achieve the related Sustainable Development Goals by 2030 (38). Food insecurity, non-exclusive breastfeeding, low birthweight and child's age were strong predictors for undernutrition.

Despite reported improvement in household characteristics and access to health facility, the prevalence of undernutrition remains high (23). Underlying factors such as low economic productivity, high unemployment rates, power-cuts, shortage of water, low quality of healthcare and water sanitation are basic causes of undernutrition (18, 26, 39). In this study, severe household food insecurity access had three-fold increase in the odds of all three types of undernutrition. The impact is profound because nearly all the households depend on purchasing of food as the main source. It is highly likely that the COVID-19 pandemic has exacerbated food insecurity and threatened other vital pathways to good nutrition (22).

Age of the child was independent risk factors of all three types of undernutrition with higher odds of wasting and underweight in children aged 0–12 months and increased odds of stunting in children between 13–24 months. This indicates the vulnerability of children to undernutrition in the first 2 years of life. In addition, the study found that exclusive breastfeeding in the first 6 months was rare in this population and this was significantly associated with underweight (40). Other studies also reported similar findings emphasizing the need for optimal nutrition in the first 2 years of life including exclusive breastfeeding in the first 6 months and continuation of breastfeeding in the first 2 years of life as are universally recommended (37). Like other studies, we observed an association between low birthweight (below 2.5 kg) and underweight (3, 9) which also highlights the need to address maternal and pregnancy-related causes of low birthweight, such as prematurity, intrauterine growth restriction, infection and adequate maternal nutrition and antenatal care as a strategy to reducing undernutrition (41).

Contrary to known evidence, secondary education was associated with increased odds of stunting as compared to caregiver with no education (3). Higher maternal education is believed to lead to better employment and financial security and thus improve household food security, good dietary diversity, better feeding practices, and healthcare access. Our results may indicate that within the current Comorian context, higher education might not be particularly advantageous because mothers who are educated are less likely to stay at home as they go out to work which affects feeding practices such as quality of meals, frequency of feeding and importantly minimizes the time and ability to breastfeed (42–44). Moreover, high unemployment rates, low and irregular salaries offer households little financial opportunity to improve food security and diversity. However, further studies need to qualitatively explore and explain why secondary education of caregivers is associated with stunting.

Since the study had a cross-sectional design, causal inference might not be strong between the outcome and independent variables. Limitations of the study include the likelihood of recall bias as caregivers might not have been able to remember information adequately and accurately. Also, a cluster sampling technique provides a homogenous study sample which could lead to bias. Furthermore, we did not back-translate the script to English. This could have led to unintended translation. However, the investigator who supervised the overall translation process is fluent both in English and Shi-Comore language and tried to avoid the fatal misunderstandings especially since Shi-Comore is not a written language.

Monitoring systems are crucial for assessing progress and identify gaps. However, data on nutritional status of children in Comoros is very scarce and outdated. Our results show possible reduction of stunting of about a third, and to lesser extent a reduction in the prevalence of underweight. In contrast wasting may have increased. These mixed results show persistently high burden of undernutrition and insufficient progress to meeting the goals of reducing undernutrition. However, since our study was only conducted in an urban region, current data from all regions of the country are needed to be certain of the observed trends. It is thus crucial to strengthen monitoring systems including a regular and timely national demographic and health survey to assess the health needs of the people in general. Further research is necessary for understanding the dynamics of child undernutrition in Comoros including underlying causes and context-informed interventions.

## Conclusion

In conclusion, this study showed a persistently high prevalence of stunting, wasting and underweight among children under-five years in Bambao region, Comoros. According to this study, food insecurity, non-exclusive breastfeeding, birthweight and child's age were strong predictors for undernutrition and the study shows the vulnerability to undernutrition in the first 2 years of life. Effective nutrition-sensitive and specific interventions using multi-sectoral approaches should be implemented at national level to address these important determinant**s** including education to mothers and caregivers about nutrition and care during pregnancy and infant and young child feeding best practices and their importance to child growth and development. Also, emphasis should be made on improving food security at national level through targeted interventions such as food programs and horizontal interventions to improve households' socio-economic standing.

## Data Availability Statement

The raw data supporting the conclusions of this article will be made available from the corresponding author upon request.

## Ethics Statement

The studies involving human participants were reviewed and approved by Muhimbili University of Health and Allied Sciences' Institutional Research and Ethics Committee IRB: MUHAS-REC-06-2021-711. Written informed consent to participate in this study was provided by the participants' legal guardian/next of kin.

## Author Contributions

HA prepared the research proposal including research questions, designed the study, planning, conducted the fieldwork, analyzed and interpreted data, and prepared the manuscript draft. BS was involved in research proposal development and supervision and revision of the manuscript. All authors read and approved the final manuscript draft.

## Conflict of Interest

The authors declare that the research was conducted in the absence of any commercial or financial relationships that could be construed as a potential conflict of interest.

## Publisher's Note

All claims expressed in this article are solely those of the authors and do not necessarily represent those of their affiliated organizations, or those of the publisher, the editors and the reviewers. Any product that may be evaluated in this article, or claim that may be made by its manufacturer, is not guaranteed or endorsed by the publisher.
