# Supplementary material for: The Burden of Undernutrition and Its Associated Factors Among Children Below 5 Years of Age in Bambao Region, Comoros
Source: Front Nutr. 2022 Apr 26;9:885002. doi: 10.3389/fnut.2022.885002 (PMC9089165; doi:10.3389/fnut.2022.885002)
Supplement: Supplementary file 1 [file Table_1.DOCX]

**Table 1:** Feeding practices among children aged 0 – 59 months in Bambao region, July 2021 (N=837).

| **Variables** | **Total** | | **Male** | | **Female** | | **p-value** |
| --- | --- | --- | --- | --- | --- | --- | --- |
|  | **N** | **%** | **N** | **%** | **N** | **%** |  |
| **Child ever breastfed**  Yes  No | 827  10 | 98.8  1.2 | 376  4.0 | 98.9  1.1 | 451  6.0 | 98.7  1.3 | 0.730 |
| **Breastfeeding initiation time**  Within an hour  After few hours | 499  338 | 59.6  40.4 | 226  243 | 59.5  40.6 | 273  184 | 273  157.4 | 0.268 |
| **Exclusive breastfeeding (n=822)**  Exclusively breastfed  Non-exclusively breastfed | 437  385 | 53.2  46.8 | 237  211 | 52.9  47.1 | 200  174 | 53.5  46.5 | 0.870 |
| **Complementary food initiation**  < 6 months  ≥ 6 months | 556  281 | 66.4  33.6 | 253  128 | 66.4  33.6 | 303  153 | 66.4  33.6 | 0.989 |
| **Continuation of breastfeeding (n=733)**  < 24 months  ≥ 24 months | 731  2 | 99.7  0.3 | 33.2  1 | 99.7  0.3 | 399  1 | 99.8  0.3 | 1.000 |
| **Child feeding frequency (24 hours)**  ≤ 3 times  ≥ 4 times | 545  292 | 65.1  34.9 | 248  132 | 65.3  34.7 | 297  160 | 65.0  35.0 | 0.934 |
| **Dietary diversity score**  < 4 scores  ≥ 4 scores | 165  672 | 19.7  80.3 | 76  305 | 19.9  80.1 | 89  367 | 19.5  80.5 | 0.876 |

**Table 2:** Healthcare factors and illnesses of children aged 0 – 59 months in Bambao region, July 2021 (N=837).

| **Variables** | **Total** | | **Male** | | **Female** | | **p-value** |
| --- | --- | --- | --- | --- | --- | --- | --- |
|  | **N** | **%** | **N** | **%** | **N** | **%** |  |
| **Antenatal care visit (n=806)**  < 4 times  ≥ 4 times | 303  503 | 37.6  62.4 | 138  228 | 37.7  62.3 | 165  275 | 37.5  62.5 | 0.748 |
| **Child vaccination**  Completed vaccination  Not completed vaccination  On vaccination routine | 645  40  152 | 77.1  4.8  18.2 | 289  15  76 | 76.1  3.9  20.0 | 356  25  76 | 77.9  5.5  16.6 | 0.302 |
| **Hospitalization in past 1 month (n=836)**  Yes  No | 113  723 | 13.5  86.4 | 48  331 | 12.6  85.8 | 65  392 | 14.2  85.8 | 0.442 |
| **Suffered from any illnesses**  No  Yes | 386  451 | 46.1  53.9 | 175  206 | 45.9  54.1 | 211  245 | 46.3  53.7 | 0.922 |
| **Diarrhea**  No  Yes | 730  107 | 87.2  12.8 | 334  47 | 87.7  12.3 | 396  60 | 86.8  13.2 | 0.723 |
| **Malaria**  No  Yes | 769  68 | 91.9  8.1 | 342  39 | 89.8  10.2 | 427  29 | 93.6  6.4 | 0.041 |
| **Cough**  No  Yes | 697  140 | 83.3  16.7 | 315  66 | 82.7  17.3 | 382  74 | 83.8  16.2 | 0.673 |
| **Vomiting**  No  Yes | 732  105 | 87.5  12.5 | 332  49 | 87.1  12.9 | 400  56 | 87.7  12.3 | 0.801 |
| **Fever**  No  Yes | 667  170 | 79.7  20.3 | 301  80 | 79.0  21.0 | 366  90 | 80.3  19.7 | 0.652 |
| **Skin diseases**  No  Yes | 801  36 | 95.7  4.3 | 367  14 | 96.3  3.7 | 434  22 | 95.2  4.8 | 0.414 |
| **Oral diseases**  No  Yes | 813  24 | 97.1  2.9 | 370  11 | 97.1  2.9 | 443  13 | 97.1  2.9 | 0.975 |
